# Pharmacokinetic and pharmacodynamic similarity evaluation between an insulin glargine biosimilar product and Lantus^®^ in healthy subjects: Pharmacokinetic parameters of both parent insulin glargine and M1 were used as endpoints

**DOI:** 10.3389/fphar.2022.962201

**Published:** 2022-08-26

**Authors:** Yiya Wang, Ying Zhou, Juefang Ding, Xianjing Li, Fengxue Guo, Jianfei Zhang, Li Ding

**Affiliations:** ^1^ Department of Pharmaceutical Analysis, China Pharmaceutical University, Nanjing, China; ^2^ Nanjing Clinical Tech Laboratories Inc., Nanjing, China; ^3^ Beijing Fosun Pharmaceutical Technology Development Co., LTD, Beijing, China; ^4^ Nanjing Jiening Pharmaceutical Technology Company, Nanjing, China; ^5^ The Second Affiliated Hospital of Xingtai Medical College, Xingtai, China; ^6^ Nanjing Yingfeng Pharmaceutical Technology Company, Nanjing, China

**Keywords:** clamp study, insulin glargine, pharmacokinetics, pharmacodynamics, biosimilarity, biosimilar product

## Abstract

Insulin glargine is a long-acting insulin analog, which plays an important role in the treatment of diabetes mellitus. Biosimilar products of insulin glargine can provide patients with additional safe, high-quality, and potentially cost-effective options for treating diabetes. This article presents a randomized, double-blind, single-dose, two-treatment, four-period, replicate crossover, euglycemic clamp study which was designed to evaluate the PK and PD similarity between the recombinant insulin glargine developed by Wanbang (test) and Lantus^®^ (reference) in healthy volunteers. Subjects received subcutaneous administration of the insulin glargine formulation (0.4 U/kg) on two occasions for the test and reference drug, respectively, and a 20% dextrose solution was infused at variable rate to clamp the blood glucose concentrations at 0.3 mmol/L below the subjects’ fasting glucose for 24 h. Taking advantage of the improved sensitivity of the bioanalytical method applied and the solution of the matrix stability problem, the parent insulin glargine was determined in the vast majority of plasma samples using a fully validated UHPLC-MS/MS method. The PK characteristics of the parent insulin glargine were revealed for the first time: after subcutaneous injection, concentrations of the parent insulin glargine increased to a relative high level within 3 h, and then, a relatively flat concentration–time profile lasting for at least 12 h post-dose was observed. For the first time, the pharmacokinetic parameters of the parent insulin glargine were used as endpoints for similarity evaluation, which complied with the regulatory guidance better and made the similarity conclusion more powerful. The ratios of geometric means of all PK and PD endpoints were close to 100.00%. For the PK endpoints (AUC_0–24h_, C_max,_ AUC_0–12h_, and AUC_12–24h_ of the parent insulin glargine and its metabolite M1), the 90% confidence intervals of geometric mean ratios of test to reference were entirely contained within 80.00%–125.00%. For the PD endpoints [AUC_GIR(0–24h)_, GIR_max,_ AUC_GIR(0–12h)_, and AUC_GIR(12–24h)_], the 95% confidence intervals of geometric mean ratios of test to reference were entirely contained within 80.00%–125.00%. Based on the above mentioned results, it can be concluded that the PK and PD characteristics of the biosimilar drug developed by Wanbang are similar to those of Lantus.

## 1 Introduction

Diabetes as a serious human health “killer” has attracted much attention (Emerging Risk Factors Collaboration et al., 2010; Bourne et al., 2013; Saran et al., 2015; United States Centers for Disease Control and Prevention, 2022). The damage of diabetes and its complications to target organs (heart, blood vessels, eyes, brain, kidney, eyes, feet, and nerves) are a serious threat to human health and quality of life (World Health Organization, 2021).

The treatment of diabetes is a worldwide problem. The emergence of insulin glargine provides a new and more advantageous tool to solve this problem because of its more stable and prolonged pharmacokinetic (PK)/pharmacodynamic (PD) profiles (Lepore et al., 2000; Scholtz et al., 2005) and its higher probability of reaching target HbA1c level without hypoglycemic events and with less glycemic variability (Perez-Maraver et al., 2013; Rys et al., 2015). First developed by Sanofi, insulin glargine (registered trademark: Lantus^®^) was approved by the US FDA and EMA in 2000 indicated to improve glycemic control in adults and children with type 1 diabetes mellitus (T1DM) and in adults with type 2 diabetes mellitus (T2DM).

In recent years, however, increases in the prices of insulin products have raised serious concerns about the ability for many patients to access the insulin needed to survive (Perez-Maraver et al., 2013; Godman et al., 2021; Saeed et al., 2022). Biosimilar products of insulin glargine can provide patients with additional safe, high-quality and potentially cost-effective options for treating diabetes (United States Food and Drug Administration, 2021b).

Biosimilar products should undergo a rigorous evaluation to demonstrate biosimilarity to the reference products before approval (European Medcine Agency, 2015; United States Food and Drug Administration, 2016; China Center for Drug Evaluation, 2021). Comparative evaluation of PK and PD profiles by means of a euglycemic clamp is the fundamental basis of assessing similarity between the biosimilar products of insulin glargine and the reference drug with respect to efficacy.

After subcutaneous injection, insulin glargine undergoes rapid transformation in the subcutaneous tissue to form two active metabolites, M1 and M2 (Kuerzel et al., 2003; Bolli et al., 2012; Lucidi et al., 2012). Due to the sensitivity limitation of analytical methods, the parent drug was not detectable in the plasma of most subjects at any dose of glargine in previous studies (Bolli et al., 2012; Lucidi et al., 2012). Therefore, previous PK evaluation was based on the active, principal component (M1) of insulin glargine (Bhatia et al., 2018; Crutchlow et al., 2018; Heise et al., 2020). However, both FDA and NMPA guidance recommended to use the parent drug to evaluate the biosimilarity because the drug concentration–time curve of the parent drug is more sensitive to changes in formulation performance than a metabolite, which is more reflective of metabolite formation, distribution, and elimination (United States Food and Drug Administration, 2021a; China Center for Drug Evaluation, 2016). A high clamp quality is important to precisely and reproducibly describe the time-action profiles of insulin analogs. The oscillation of blood glucose concentration can result in insufficient suppression of endogenous insulin and affect the accuracy of pharmacodynamics data (Benesch et al., 2015; Tao et al., 2021). Also, it is reported that the degree of inhibition of endogenous insulin secretion is associated with glucose oscillations, PK/PD assessment, and the quality of clamp study (Tao et al., 2021). However, the quality assessment method of clamp studies reported in the previous articles is varied but not comprehensive (Bhatia et al., 2018; Crutchlow et al., 2018). A previous study reported that due to the high intra-subject variability of PK and PD endpoints of insulin glargine, large sample size and even replicate crossover study design were necessary to provide at least 80% overall power to meet all hypotheses (Scholtz et al., 2005; Bhatia et al., 2018; Crutchlow et al., 2018). The intra-subject variability of the PK and PD endpoints in Chinese healthy volunteers has not been reported.

Recombinant insulin glargine is a biosimilar product developed by Wanbang Biopharmaceuticals. Clinical evaluations of this biosimilar product of insulin glargine to meet the requirements of the China National Medical Products Administration for biosimilarity are currently underway. A phase III study in subjects with T2DM has been conducted and successfully demonstrated the similarity with regard to safety, immunogenicity, and efficacy outcomes.

This article reported a clamp study which was designed to evaluate the similarity of the PK and PD properties of the recombinant insulin glargine (test) developed by Wanbang and Lantus (reference) in healthy volunteers. Taking advantage of the improved sensitivity of the bioanalytical method applied and the solution of matrix stability problem, the PK characteristics of the parent insulin glargine were revealed; thus, PK parameters of the parent insulin glargine together with M1 were used as endpoints for similarity evaluation for the first time.

## 2 Materials and methods

### 2.1 Study design

This PK/PD comparative study was a randomized, double-blind, single-dose, two-treatment, four-period, replicate crossover, euglycemic clamp study. Subjects were randomly assigned to one of two dosing sequences and received 0.4 U/kg reference drug on two occasions and 0.4 U/kg test drug on two occasions. The dosing nurses were not blind to the treatment allocation, whereas the subjects and the clamping operators (who adjusted the glucose infusion rate) were blinded.

Subjects were admitted to the clinical research unit on the night before each period. On day 1 of each period, all doses of test and reference drug were administered by subcutaneous injection, and then, subjects underwent a euglycemic clamp procedure until 24 h post-dose. There was a washout period of 10–14 days between study periods.

The study was performed in accordance with the ICH-GCP guidelines and the Declaration of Helsinki. The study protocol was approved by the Independent Ethics Committee of The Second Affiliated Hospital of Xingtai Medical College (No. XYERCTEC-HS-060). All subjects provided written informed consent forms (ICFs).

### 2.2 Study subjects

Healthy Chinese male volunteers aged from 18 to 45 and with a body mass index between 19.0 and 24.0 kg/m^2^ were eligible for recruitment. The health of the subjects was assessed at the screening visit, which included a medical history, measurement of body weight and height, recording of vital signs, a physical examination, chest X-ray, 12-lead ECG, clinical laboratory tests (hematology, blood biochemistry, urinalysis, coagulation function, fasting blood glucose, 2 h oral glucose tolerance test, hepatitis B and C, HIV, and treponema pallidum), smoking, breath alcohol test, drugs abuse, and medications taken during the 2 weeks preceding the screening visit.

### 2.3 Study drugs and dose

The test drug, manufactured by Wanbang Biopharmaceuticals, was supplied as a 100 U/ml solution in 3 ml cartridges. The reference drug was procured commercially as cartridges. The test drug or reference drug was administrated by subcutaneous injection using a pen about 5 cm from the left or right side of the umbilicus, as a 0.4 U/kg dose.

### 2.4 Euglycemic clamp procedure

For each subject, the 24 h euglycemic clamp procedure aimed to maintain the clamp value at 0.3 mmol/L below the subject’s mean fasting glucose level in each period. Each subject began fasting from 20:00 on the day prior to dosing in each period and continued fasting during the clamp study. Body weight and vital signs were measured after the bladder was emptied, and the clamp study was performed after resting for at least 30 min. One upper limb was cannulated for the infusion of glucose (20% dextrose solution in water). The contralateral upper limb was cannulated for PK blood sample collection and blood glucose concentration measurement. Blood glucose level was measured by using an automatic blood glucose analyzer every 20 min from 0 to 3.0 h, every 10 min from 3.0 to 12.0 h, every 15 min from 12.0 to 16.0 h, every 30 min from 16.0 to 21.0 h, and every 15 min from 21.0 to 24.0 h. After each glucose measurement, the glucose infusion rate (GIR) was manually adjusted by the operator based on the experience of the change rate of blood glucose to maintain the blood glucose concentration within ±10% of the clamp target. During the entire procedure, subjects should stay awake, decumbent, or semi-decumbent as much as possible and relieve themselves in time with the help of medical staff.

### 2.5 Pharmacokinetic sampling

In each period, serial blood samples for the quantification of insulin glargine, its metabolites (M1 and M2), and C-peptide were collected at 30 min prior to dosing and 0, 1, 3, 5, 6, 7, 8, 9, 10, 11, 12, 13, 14, 16, 18, 21, and 24 h after dose using vacuum tubes with an EDTA anticoagulant.

### 2.6 Bioanalytical methods

Concentrations of insulin glargine and its metabolites (M1 and M2) in plasma were simultaneously quantified by an ultra-high-performance liquid chromatography tandem mass spectrometry (UHPLC-MS/MS) method developed and fully validated before the clinical sample analysis. Insulin glargine, M1, M2, and the internal standard (bovine insulin) were extracted by MCX solid-phase extraction on an ice-bath prior to injection into the UHPLC-MS/MS system using 250 μl of plasma. Chromatographic separations were performed with Shimadzu LC-30AD (Shimadzu, Kyoto, Japan) on a CORTECS^®^ UPLC^®^ C18+ column (2.1 mm × 50 mm; 1.6 μm) (Waters, Milford, Massachusetts, United States). Mass spectrometric detection was performed with Triple Quad™ 6500+ (AB SCIEX, Foster City, California, United States) operating in the electron spray ionization positive ion mode. The method was linear over the concentration ranges of 75.0–1,000, 125–1,000, and 125–1,000 pg/ml for insulin glargine, M1, and M2, respectively. Details of this assay are available in the Supplementary Material.

C-peptide in plasma was quantified by a fully validated enzyme-linked immunosorbent assay (ELISA) method, of which the calibration range was 28.9–925 pmol/L.

### 2.7 Pharmacokinetic assessment

PK comparison in this study was based on the principal component M1, as well as the parent insulin glargine. Pharmacokinetic parameters were calculated with WinNonlin Version 8.2 (Pharsight Corporation, Mountain View, California, United States) using a non-compartmental analysis method. The primary PK parameters were maximum plasma concentration (C_max_) and area under the plasma concentration–time curve (AUC_0–24 h_). Partial area under the plasma concentration–time curve (AUC_0–12 h_ and AUC_12–24 h_) was a secondary PK parameter. Time to reach C_max_ (T_max_) was also calculated.

C-peptide concentration was measured in parallel to insulin concentrations throughout the experiment to compare the extent and consistency of suppression of endogenous insulin between the two drugs.

### 2.8 Pharmacodynamic assessment

PD parameters were calculated with WinNonlin Version 8.2 using the trapezoidal rule. Raw GIR profiles were not smoothed before calculation of PD parameters. Area under the glucose infusion rate–time curve (AUC_GIR(0–24 h)_) and peak of glucose infusion rate (GIR_max_) were measured as primary endpoints. Partial area under the glucose infusion rate–time curve (AUC_GIR(0–12 h)_ and AUC_GIR(12–24 h)_) was the secondary PD parameter. Another meaningful PD endpoint was time to GIR_max_.

### 2.9 Safety assessment

The subjects were under continuous medical supervision in The Second Affiliated Hospital of Xingtai Medical College. Prior to dose and at various time points post-dose and post-study, assessments of safety and tolerability were conducted by monitoring adverse events (AEs), vital signs, physical examinations, laboratory tests (hematology, blood biochemistry, urinalysis, and coagulation function), and 12-lead ECG.

### 2.10 Statistical analysis

#### 2.10.1 Quality estimation of the clamps

The quality of the performance of the clamp study was estimated from two aspects. On one hand, the blood glucose level during the entire clamping process was evaluated by calculating the mean difference between the blood glucose concentration and the clamp target, mean square deviation, and coefficient of variation of the blood glucose concentrations. On the other hand, the plasma C-peptide concentration profiles and the ratio of C-peptide reduction were evaluated.

#### 2.10.2 Pharmacokinetics

The primary and secondary PK parameters, AUC_0–24 h_, C_max_, AUC_0–12 h_, and AUC_12–24 h_, were log transformed prior to analysis. A linear mixed-effects model was used for statistical comparison, which included subject as a random effect with period, sequence, and treatment as fixed effects. For each PK parameter, the difference in geometric means along with the 90% confidence intervals (CIs) was back transformed to produce the ratio of geometric means and the CI comparing the test drug to reference drug. Similarity was to be concluded if the 90% CIs for all PK parameters were contained within the interval of 80.00%–125.00%. Intra-subject and inter-subject variability were reported for each PK parameter.

#### 2.10.3 Pharmacodynamics

The primary and secondary PD parameters, AUC_GIR(0–24 h)_, GIR_max_, AUC_GIR(0–12 h)_, and AUC_GIR(12–24 h)_, were log transformed prior to analysis. The same linear mixed-effects model as PK comparison was used for the PD comparison. For each PD parameter, the difference in geometric means along with the 90% CIs was back transformed to produce the ratio of geometric means and the CI comparing the test drug to reference drug. PD similarity was to be concluded if the 95% CIs for both primary and secondary PD parameters were contained within the interval of 80.00%–125.00%. Intra-subject and inter-subject variability were reported for each primary and secondary PD parameters.

## 3 Results

### 3.1 Study population

A total of 190 subjects were screened, and finally, 38 healthy male subjects were enrolled into the study. All the enrolled participants completed the study according to the protocol. The demographic details (mean ± SD) were age 27.5 ± 5.7 years, weight 62.3 ± 5.3 kg, height 170.1 ± 4.6 cm, and body mass index 21.5 ± 1.2 kg/m^2^.

### 3.2 Quality of the clamps

In this clamp study, blood glucose concentrations were maintained very close to the clamp targets with a coefficient of variation of 5.0% and 4.8% for the test and reference drug, respectively. The detailed evaluation results of blood glucose concentrations are shown in the Supplementary Material.

**FIGURE 1:**
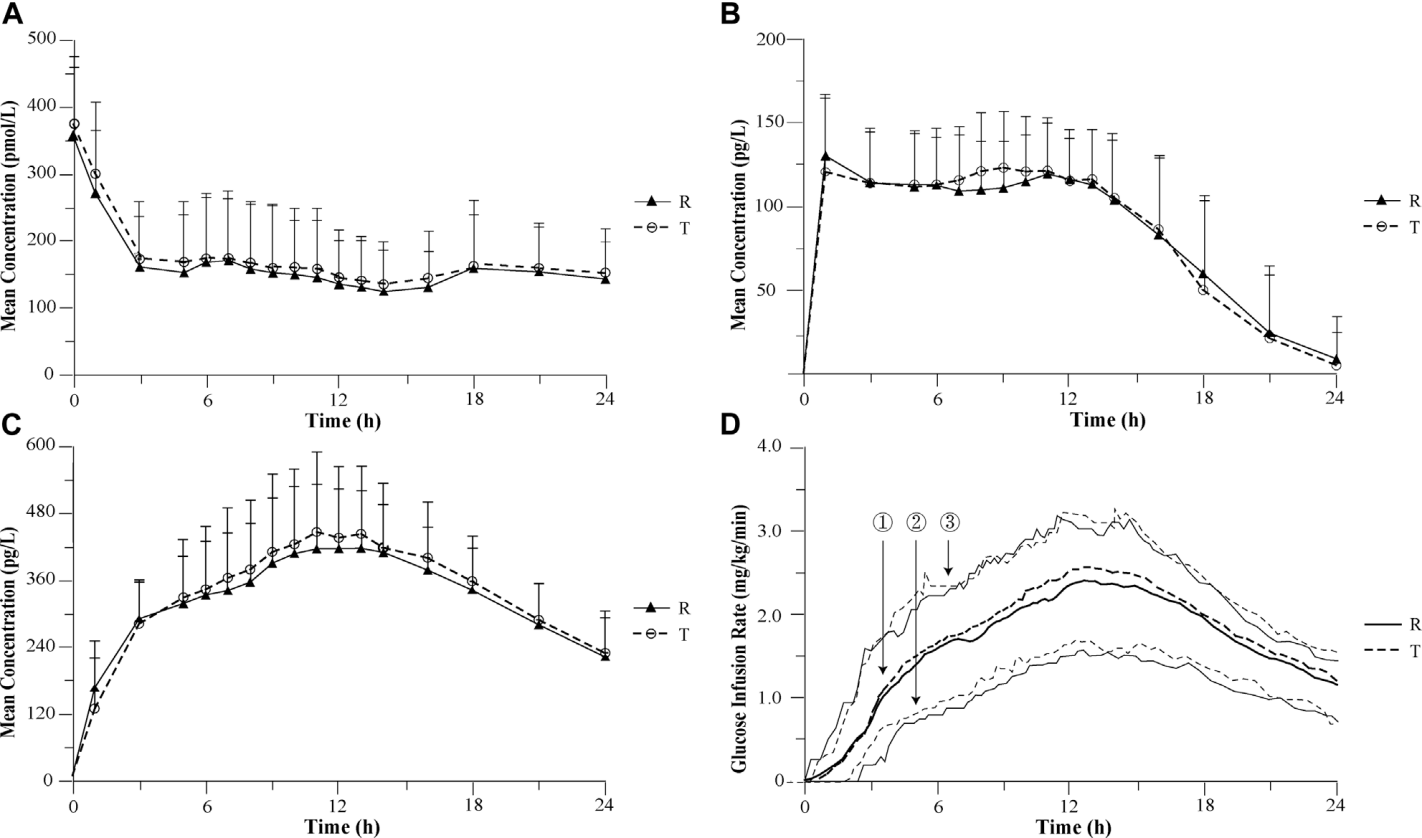
Mean pharmacokinetic and pharmacodynamic profiles after 0.4 U/kg doses of recombinant insulin glargine developed by Wanbang and Lantus in healthy volunteers. **(A)** Mean C-peptide concentration–time profiles after 0.4 U/kg doses of recombinant insulin glargine developed by Wanbang and Lantus in healthy volunteers. **(B)** Mean parent insulin glargine concentration–time profiles after 0.4 U/kg doses of recombinant insulin glargine developed by Wanbang and Lantus in healthy volunteers. **(C)** Mean M1 concentration–time profiles after 0.4 U/kg doses of recombinant insulin glargine developed by Wanbang and Lantus in healthy volunteers. **(D)** Glucose infusion rate–time profiles after 0.4 U/kg doses of recombinant insulin glargine developed by Wanbang and Lantus in healthy volunteers. The medians (①: thick solid line for the reference drug; thick dotted line for the test drug) and the 25th and 75th percentiles (② and ③: thin solid line for the reference drug; thin dotted line for the test drug) are given.

Similar mean plasma C-peptide profiles were observed following the administration of the test and reference drug (Figure 1A). The individual plasma C-peptide profiles are shown in the Supplementary Material. The ratios of reduction C-peptide reduction were comparable between two treatments (56.5% ± 14.1% for the test drug; 57.5% ± 14.4% for the reference drug), suggesting a similar degree of suppression of the endogenous insulin following the administration of either drug.

### 3.3 Pharmacokinetics

The parent insulin glargine was determined in the vast majority of plasma samples, and concentration–time profiles with at least 12 quantifiable post-dose concentrations were obtained for a total of 34 subjects. The PK comparison of the parent insulin glargine was based on the results of these 34 subjects. Following subcutaneous injection of a single dose, the mean parent insulin glargine and M1 plasma concentration profiles were similar between the test and reference drug (Figures 1B,C). The 90% CIs of the geometric mean ratios for AUC_0–24 h_, C_max_, AUC_0–12 h_, and AUC_12–24 h_ were also entirely within the pre-specified interval of 80.00%–125.00%, demonstrating the PK similarity between the test and reference drug (Table 1). The replicate design of this study permitted assessment of intra-subject variability for PK parameters (Table 1).

**TABLE 1:** Comparison of pharmacokinetic and pharmacodynamic parameters between recombinant insulin glargine developed by Wanbang and Lantus.

	N^a^	Geometric mean		Ratio	90% or 95% CIs of the ratio^d^	Intra-subject variability (%)	
		Test drug^b^	Reference drug^c^			Test drug^b^	Reference drug^c^
PK parameters of the parent insulin glargine							
AUC_0–24 h_ (pg/ml·h)	34	1880	1920	98.24	91.35–105.65	22.1	15.9
C_max_ (pg/ml·h)	34	149	149	99.78	93.99–105.92	16.9	12.3
AUC_0–12 h_ (pg/ml·h)	34	1,320	1,310	100.56	94.82–106.65	15.5	11.7
AUC_12–24 h_ (pg/ml·h)	34	591	617	95.47	81.42–112.04	52.9	44.1
T_max_ (h)^e^	34	8 (1,16)	3 (1,16)				
PK parameters of M1							
AUC_0–24 h_ (pg/ml·h)	38	7,391	7217	102.41	96.16–109.06	20.2	15.7
C_max_ (pg/ml·h)	38	449	427	105.21	97.79–113.19	21.2	18.6
AUC_0–12 h_ (pg/ml·h)	38	3,455	3437	100.50	92.65–109.01	27.2	17.9
AUC_12–24 h_ (pg/ml·h)	38	3,884	3736	103.94	98.52–109.66	17.6	14.5
T_max_ (h)^e^	38	12 (3,24)	12 (5,18)				
Primary PD parameters							
AUC_GIR(0–24 h)_ (mg/kg/min·h)	38	36.3	34.4	105.41	96.78–114.81	36.4	28.3
GIR_max_(mg/kg/min)	38	2.66	2.54	105.00	97.99–112.50	26.5	25.5
AUC_GIR(0–12 h)_ (mg/kg/min·h)	38	13.52	13.45	100.52	84.88–119.05	70.7	39.0
AUC_GIR(12–24 h)_ (mg/kg/min·h)	38	22.45	21.19	105.91	97.96–114.51	27.3	20.2
Time to GIR_max_ ^e^	38	12.3 (3.85, 21.5)	11.8 (4, 19.0)				

a N, number of subjects.

b Test drug: recombinant insulin glargine developed by Wanbang.

c Reference drug: Lantus.

d For PK parameters, 90% CIs of the ratio are displayed; for PD parameters, 95% CIs of the ratio are displayed.

e T_max_ and time to GIR_max_ are displayed as median (range).

### 3.4 Pharmacodynamics

Following a single subcutaneous administration, the median and inter-quartiles of the GIR-time profiles were essentially overlapping (Figure 1D). The statistical comparison of AUC_GIR(0–24 h)_,GIR_max_, AUC_GIR(0–12 h)_, and AUC_GIR(12–24 h)_ demonstrated similarity in PD between the test and reference drug (Table 1). The ratios of geometric means were 105.41, 105.00, 100.52, and 105.91 for AUC_GIR(0–24 h)_, GIR_max_, AUC_GIR(0–12 h)_, and AUC_GIR(12–24 h)_, respectively, with the 95% CIs for the ratios contained with the pre-specified interval of 80.00%–125.00%. The intra-subject variability for PD parameters was also assessed for both treatments (Table 1).

### 3.5 Safety evaluation

The safety profiles of recombinant insulin glargine and Lantus were comparable with regard to AEs, and there were no changes in the vital signs, physical examinations, laboratory tests (hematology, blood biochemistry, urinalysis, and coagulation function), or 12-lead ECG parameters during the study that were considered clinically significant by the investigator.

## 4 Discussion

### 4.1 Similarity evaluation

Recombinant insulin glargine is a biosimilar of Lantus and is developed to improve glycemic control. The present study provides assessment of PK and PD similarity between recombinant insulin glargine developed by Wanbang and Lantus after a single 0.4 U/kg subcutaneous injection in healthy subjects. This study was designed to comply with available regulatory guidance for the development of biosimilar insulins. The evidence presented by this study supported that the PK and PD (time-action) profile of the test drug was similar to that of the reference drug. The PK and PD similarity was adequately demonstrated.

### 4.2 Study design

Healthy volunteers rather than patients with T1DM were recruited into this study based on the following considerations. First, healthy volunteers are more available. Second, healthy volunteers usually exhibit lower intra-individual variability compared to patients with T1DM. Third, the UHPLC-MS/MS assay used in this study can distinguish exogenous insulin (insulin glargine and its metabolites) from endogenous insulin. Last but not the least, the method for suppressing endogenous insulin was carried out by clamping the blood glucose concentrations at 0.3 mmol/L below the subjects fasting glucose levels, which could reduce the interference of the endogenous insulin to PD results. If the endogenous insulin is well suppressed with high ratios of C-peptide reduction and the suppression is comparable between the test drug and reference drug, the low contribution of the endogenous insulin to the PD results does not affect the reliability of the PD similarity evaluation results.

The setting of the sampling scheme for blood glucose measurement was based on the change rates of the blood glucose concentration levels during the entire clamp process. The change rates were obtained from the pilot clamp study. In addition, the total insulin glargine (insulin glargine and its metabolites) plasma concentration–time profiles and glucose infusion rate–time profiles obtained from the pilot study and those reported in previous studies were also referenced. During the periods of the highest exposure to the total insulin glargine and the time to GIR_max_, the sampling intervals were set to 10 min; when the exposure levels were low, the sampling intervals were set to be slightly longer. According to the quality assessment results of the clamps, the blood glucose concentrations were maintained very close to the clamp targets (0.3 mmol/L below the subjects fasting glucose levels). These results indicate the setting of the sampling intervals of this study is suitable.

Due to ethics considerations (subject tolerance) and the insulin glargine administration instructions (once per day), the duration of this clamp study was set to 24 h, which was the same as that in the previous reported studies in healthy volunteers (Bhatia et al., 2018; Crutchlow et al., 2018). Although this duration did not allow the full capture of the PK characteristics of M1, it complied with the EMA guidance (European Medcine Agency, 2015), and it was sufficient for similarity evaluation.

### 4.3 Pharmacokinetic characteristics of the parent insulin glargine and its application to the similarity evaluation

The bioanalytical method applied in this study was with a relatively higher sensitivity of 75.0 pg/ml for the parent insulin glargine (Bhatia et al., 2018; Crutchlow et al., 2018). The instability of the insulin glargine in whole blood and plasma was addressed by strict temperature control and selection of EDTA anticoagulant during sample collection and pre-treatment. Thus, the parent insulin glargine was determined in the vast majority of plasma samples, and the PK characteristics of parent insulin glargine in healthy subjects were revealed for the first time. After subcutaneous injection, the concentrations of parent insulin glargine increased to a relatively high level within 3 h, and then, a relatively flat concentration–time profile lasting for at least 12 h post-dose was observed, which implied a prolonged absorption of insulin glargine. The key pharmacokinetic parameters of the parent insulin glargine together with M1 following subcutaneous injection of a single dose were calculated and applied to demonstrate the PK similarity of the test and reference drugs for the first time.

Modification of the basic insulin structure makes the insulin glargine soluble at an acidic pH, but precipitates in the subcutaneous tissue after injection to form a depot (Levien et al., 2002). Then, the drug is slowly released from the depot. This explains why flat concentration–time profiles of parent insulin glargine were observed in this study. Compared with the main metabolite M1, the parent insulin glargine showed an earlier T_max_ and a flat concentration–time profile. These observed phenomena are consistent with those of a previous study (Bolli et al., 2012). Based on the comparison of AUC and C_max_, the exposure of the parent insulin glargine was about 1/3 that of M1, indicating that parent insulin glargine and M1 are the dominant circulating insulin glargine species after subcutaneous injection and they mainly mediate the PD effect of insulin glargine. Insulin glargine exhibits a flat PK profile, and as a long-acting insulin, the difference in the T_max_ values between the test and reference drugs is clinically meaningless (European Medcine Agency, 2015). The drug concentration–time curve of the parent drug is more sensitive to changes in formulation performance than a metabolite, which is more reflective of metabolite formation, distribution, and elimination (United States Food and Drug Administration, 2021). Therefore, applying the PK comparison of the parent insulin glargine together with M1 to the similarity evaluation makes the conclusion more powerful. Finally, the PK comparison results of the parent insulin glargine together with M1 were submitted to the China National Medical Products Administration to support the application of the test drug.

### 4.4 Quality assessment of clamp studies

The clamping precision of this study is of the same degree of other similar studies (Benesch et al., 2015; Heise et al., 2015; Crutchlow et al., 2018), and this demonstrates that this clamp study was of a high degree of precision. Also, in this study, the ratios of C-peptide reduction were above 50.0%, demonstrating that the PD results were free from the interference of endogenous insulin (Tao et al., 2021).

A high glucose clamp quality is important to precisely and reproducible describe the time-action profiles of insulin analogs. However, data on the quality of clamp study have been reported incompletely in previous studies (Bhatia et al., 2018; Crutchlow et al., 2018). The quality estimation of the performance of a clamp study includes two parts, the statistics of blood glucose concentration and plasma C-peptide comparison.

The more stable the blood glucose level maintains and the closer the glucose is maintained to the target level, the more the infusion rate can truly reflect the consumption of blood glucose, i.e., the efficacy of insulin analogs. However, there are no clear parameter requirements for the evaluation of blood glucose, so that varied, but no comprehensive parameters were applied in the previous study. In this study, the root mean square deviation and coefficient of variation of the blood glucose concentrations were calculated to show the stability of blood glucose maintenance (precision). Meanwhile, the mean difference between the actual glucose concentration and the target together with the mean value of the blood concentrations was calculated to assess the control accuracy, which shows the difference between actual blood glucose levels and target much clearly. Precision and accuracy are used to assess the quality of clamp study from different angles, and both are indispensable. A high-quality clamp study usually has a coefficient of variation of the blood glucose concentrations below 5.0% and a mean difference within 5.0%.

The comparability of endogenous insulin production between the two drugs was often demonstrated by comparing the C-peptide concentration profiles and ratio of C-peptide reduction. The C-peptide concentration profile is not a quantitative indicator, and the ratio of C-peptide reduction effectively reflects the suppression of endogenous insulin during the entire clamping process.

### 4.5 Intra-subject variability

Multiple precedent PK/PD similarity evaluation studies reported that the intra-subject variability of PK parameters (AUC_0–24 h_ and C_max_) was about 30.0%, and the intra-subject variability of PD parameters (AUC_GIR(0–24 h)_ and GIR_max_) was even greater (Scholtz et al., 2005; Bhatia et al., 2018; Crutchlow et al., 2018). Thus, a larger sample size and replicate crossover study design were necessary. This study showed a relatively lower intra-subject variability of PK parameters and PD parameters, which may be due to the fact that the healthy male subjects enrolled in this study were more homogeneous. Moreover, strategies to control the intra-subject variability in this study involve the following aspects: management of subjects, process control of clamping to improve the quality of clamp study and guarantee the accurate measurement and fast adjustment of blood glucose level, sufficient PK sampling points, and sensitivity improvement of the bioanalytical method to obtain more reliable concentration data.

## 5 Conclusion

In summary, this study demonstrated similarity in PK (AUC_0–24 h_, C_max,_ AUC_0–12 h_, and AUC_12–24 h_) and PD (GIR_AUC(0–24 h)_, GIR_max_, AUC_GIR(0–12 h)_, and AUC_GIR(12–24 h)_) characteristics of the insulin glargine biosimilar product and Lantus upon a single subcutaneous dose of 0.4 U/Kg in healthy subjects with comparable safety profiles. The PK characteristics of the parent insulin glargine were revealed, and its pharmacokinetic parameters were used as endpoints for similarity evaluation for the first time. Furthermore, a comprehensive method for the quality assessment of clamp studies and control strategy of intra-subject variability were proposed in this study, which will provide a good reference for the similarity evaluation study of insulin analogs in the future.

## Data Availability

The original contributions presented in the study are included in the article/Supplementary Material; further inquiries can be directed to the corresponding author.
