# Supplementary material for: Pharmacokinetic and pharmacodynamic similarity evaluation between an insulin glargine biosimilar product and Lantus® in healthy subjects: Pharmacokinetic parameters of both parent insulin glargine and M1 were used as endpoints
Source: Front Pharmacol. 2022 Aug 26;13:962201. doi: 10.3389/fphar.2022.962201 (PMC9459017; doi:10.3389/fphar.2022.962201)
Supplement: Supplementary file 1 [file DataSheet1.DOCX]

Supplementary Material

**Details of an UHPLC-MS/MS Method for the Simultaneous Determination of Insulin Glargine and its Metabolites in Human Plasma**

**Sample treatment procedures**

250 μL of samples were pre-treated with 375 μL of AA:MeOH:ACN (1:50:50, *v*/*v*/*v*) on ice-bath, and mixed. After centrifuge, the supernatants were then extracted using Oasis^®^ MCX μElution Plate 30 μm (Waters, Milford, Massachusetts, United States). The samples were washed with FA:H_2_O (1:50, *v*/*v*), AA:MeOH:H_2_O (1:5:100, *v*/*v*/*v*), and ACN:H_2_O (1:4, *v*/*v*) successively, and eluted in ammonium hydroxide:H_2_O:MeOH (1:6:14, *v*/*v*/*v*). The elution was evaporated to dryness under a stream of nitrogen, and then reconstituted with AA:MeOH: H_2_O (1:3:6, *v*/*v*/*v*) prior to injection onto the UHPCL-MS/MS system.

**LC conditions**

| UHPLC system: | Shimadzu LC-30AD |
| --- | --- |
| Column: | CORTECS^®^ UPLC^®^ C18+ column (2.1×50 mm; 1.6 μm) (Waters, Milford, Massachusetts, United States) |
| Column temperature: | 40 ºC |
| Flow rate: | 0.300 mL/min |
| Mobile phase A: | FA:H_2_O (1:500, *v*/*v*) |
| Mobile phase B: | FA:ACN (1:500, *v*/*v*) |
| Injection volume: | 30 μL |
| Gradient: | \| Time \| A% \| B% \| \| --- \| --- \| --- \| \| 0.00 \| 85 \| 15 \| \| 0.50 \| 85 \| 15 \| \| 2.50 \| 70 \| 30 \| \| 3.50 \| 70 \| 30 \| \| 3.60 \| 50 \| 50 \| \| 4.60 \| 50 \| 50 \| \| 4.70 \| 10 \| 90 \| \| 5.70 \| 10 \| 90 \| \| 5.80 \| 85 \| 15 \| \| 7.30 \| 85 \| 15 \| |

**MS conditions**

| MS system: | Triple QuadTM 6500+ (AB SCIEX, Foster City, California, United States) |
| --- | --- |
| Mode: | Scheduled MRM, ESI, Positive |
| Source: | IonSpray Voltage：5500 V  CUR：35 psi  GS1：50 psi  GS2：50 psi  TEM：450 °C  Resolution Q1: Unit  Resolution Q3: Unit  Pause between mass：5.0070ms |
| Collision gas: | 10 |
| MRM transitions: | \| Compound  name \| Precursor  (*m/z*) \| Product  (*m/z*) \| DP  (V) \| EP  (V) \| CE  (V) \| EP  (V) \| \| --- \| --- \| --- \| --- \| --- \| --- \| --- \| \| Insulin glargine \| 867.0 \| 984.1 \| 70 \| 10 \| 27 \| 25 \| \| M1 \| 959.4 \| 1118.4 \| 70 \| 10 \| 30 \| 35 \| \| M2 \| 942.5 \| 1098.1 \| 110 \| 10 \| 29 \| 43 \| \| Bovine insulin \| 963.9 \| 1123.8 \| 70 \| 10 \| 31 \| 20 \| |

**Supplementary Table 1 Blood glucose concentration parameters referring to clamp quality**

| Formulation | Basal blood glucose level (mmol/L) | Clamp target (mmol/L) | Mean blood glucose concentraion after dose  (mmol/L) | SD^c^ (mmol/L) | CV^d^ (%) | Mean difference^e^  (%) |
| --- | --- | --- | --- | --- | --- | --- |
| Test Drug^a^ | 4.58±0.24 | 4.28±0.24 | 4.27±0.21 | 0.21 | 5.0 | 2.99±0.52 |
| Reference Drug^b^ | 4.53±0.23 | 4.23±0.23 | 4.23±0.20 | 0.2 | 4.8 | 2.98±0.65 |

^a^Test drug: Recombinant insulin glargine developed by Wanbang.

^b^Reference drug: Lantus.

^c^SD: Standard deviation.

^d^CV: Coefficient of variation.

^e^Mean difference: Mean difference between the actual blood glucose concentration and the clamp target.

**Supplementary Figure 1 Individual plasma C-peptide profiles after 0.4 U/kg Doses of Recombinant Insulin Glargine Developed by Wanbang and Lantus in Healthy Volunteers**





(A) Individual plasma C-peptide profiles after 0.4 U/kg Doses of Recombinant Insulin Glargine Developed by Wanbang in Healthy Volunteers





(B) Individual plasma C-peptide profiles after 0.4 U/kg Doses of Recombinant Insulin Glargine Developed by Wanbang in Healthy Volunteers
